# Anti-influenza A virus activity and structure–activity relationship of a series of nitrobenzoxadiazole derivatives

**DOI:** 10.1080/14756366.2021.1982932

**Published:** 2021-09-28

**Authors:** Francesco Fiorentino, Marta De Angelis, Martina Menna, Annarita Rovere, Anna Maria Caccuri, Francesca D’Acunzo, Anna Teresa Palamara, Lucia Nencioni, Dante Rotili, Antonello Mai

**Affiliations:** a Department of Drug Chemistry and Technologies, Sapienza University of Rome, Rome, Italy; b Department of Public Health and Infectious Diseases, Laboratory Affiliated to Istituto Pasteur Italia-Fondazione Cenci Bolognetti, Sapienza University of Rome, Rome, Italy; c Department of Chemical Sciences and Technologies, University of Rome Tor Vergata, Rome, Italy; d CNR, Istituto di Metodologie Chimiche, Sezione Meccanismi di Reazione, Sapienza University of Rome, Rome, Italy; e Department of Infectious Diseases, Istituto Superiore di Sanità, Rome, Italy

**Keywords:** Influenza A virus, nitrobenzoxadiazoles, RNA polymerase inhibitors, antivirals

## Abstract

Influenza viruses represent a major threat to human health and are responsible for seasonal epidemics, along with pandemics. Currently, few therapeutic options are available, with most drugs being at risk of the insurgence of resistant strains. Hence, novel approaches targeting less explored pathways are urgently needed. In this work, we assayed a library of nitrobenzoxadiazole derivatives against the influenza virus A/Puerto Rico/8/34 H1N1 (PR8) strain. We identified three promising 4-thioether substituted nitrobenzoxadiazoles (**12**, **17**, and **25**) that were able to inhibit viral replication at low micromolar concentrations in two different infected cell lines using a haemagglutination assay. We further assessed these molecules using an In-Cell Western assay, which confirmed their potency in the low micromolar range. Among the three molecules, **12** and **25** displayed the most favourable profile of activity and selectivity and were selected as hit compounds for future optimisation studies.

## Graphical abstract

**Figure:**
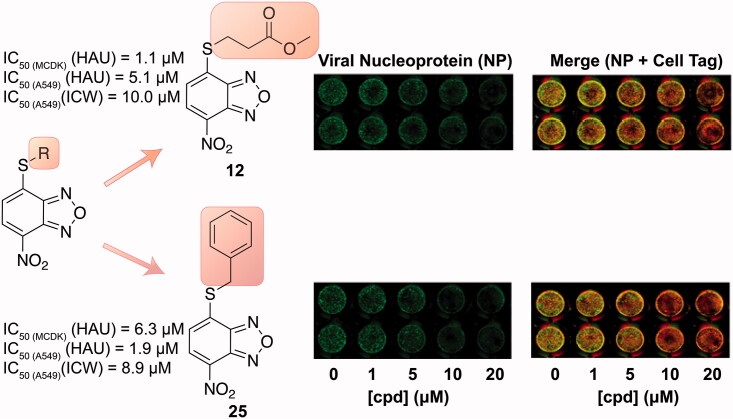

## 1. Introduction

Influenza is a respiratory infectious disease characterised by high morbidity and mortality especially in at-risk subjects. Influenza viruses (IVs) belong to the family of *Orthomyxoviridae* and are characterised by a segmented, single-stranded, negative-sense RNA genome^1^. Type A IVs infect a broad range of animals, including humans, while type B and C IVs are predominantly human pathogens^2^. Type A and B IVs are both responsible for seasonal epidemics; in addition, type A IV has caused pandemics in the past leading to millions of deaths and hospitalisation. These include the 1918–1920 “Spanish” flu^3^ and the 2009 (H1N1) pandemic^4^^,^^5^ which was the result of a triple reassortment of bird, swine, and human IVs that further combined with a Eurasian pig IV^6^, leading to the term “swine flu”.

**Figure 1.**
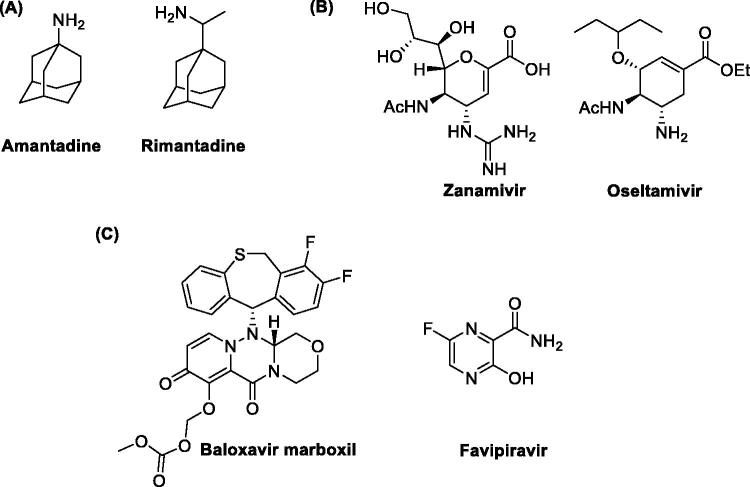
Most representative commercially available anti-IV drugs.

The therapeutic options currently available for IV infections include vaccination and antiviral drugs used to prevent and treat them, respectively^7^^,^^8^. To date, three classes of antivirals have been approved for clinical use: (i) the adamantanes, (ii) the inhibitors of the viral glycoprotein Neuraminidase (NA) and (iii) the compounds targeting the viral RNA-dependent RNA polymerase (RdRp), which represent the latest therapeutic approach to combat IVs. Adamantanes are the oldest and most affordable molecules to treat IVs and include amantadine and rimantadine (Figure 1(A))^9^. These compounds target the viral ion-channel Matrix (M2) protein required for virus uncoating^10–12^. However, both molecules possess potentially serious central nervous system side effects and nowadays most IV strains are resistant to these drugs. NA inhibitors impair the release of viral particles from infected host cells. Among them, the most used drugs are zanamivir and oseltamivir (Figure 1(B))^13^^,^^14^ which have an improved safety profile compared to adamantanes. Nonetheless, the efficacy of these antivirals is often limited by the high antigenic variability of the virus,the huge circulation of different strains, and the development of resistance to the available drugs.

Given this situation, many lines of research have been directed towards the discovery of novel anti-influenza drugs. To this end, the RdRp has been identified as an interesting target for the inhibition of viral replication because it is an element conserved among different viral strains and is not subjected to great genetic variability^15^. Drug discovery efforts finally led to two RdRp inhibitors recently approved for clinical use: baloxavir marboxil and favipiravir (Figure 1(C)). Baloxavir marboxil is a prodrug of baloxavir acid (Figure 1(C)), an inhibitor of the cap-dependent endonuclease activity of RdRp of both type A and B IVs^16^. Favipiravir (Figure 1(C)), following activation to the corresponding ribofuranosyl-5′-triphosphate, acts as a purine nucleoside triphosphate analogue and it is thus recognised as an alternative substrate by the RdRp^17^. Baloxavir marboxil has been licenced for the treatment and prevention of influenza in different countries, including Japan^18^^,^^19^, United States of America^20^^,^^21^, and European Union^22^, while favipiravir is only licenced in Japan^23^ for the emergency treatment of IV strains insensitive to current antivirals. These successful examples highlight the potential of RdRp as a drug target and, beyond marketed drugs, different groups have been researching novel inhibitors targeting either its enzymatic activity or its structural integrity.

**Figure 2.**
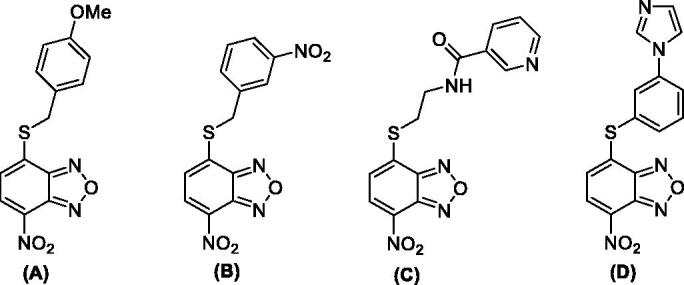
Most relevant benzoxadiazole derivatives (A–D) previously reported as anti-IV A agents^24^^,^^25^.

The enzyme RdRp is a heterotrimeric complex, consisting of three proteins: polymerase basic protein 1 (PB1), polymerase basic protein 2 (PB2), and polymerase acidic protein (PA). The three subunits form a multi-protein complex, with PB1 as the central core interacting with PB2 on one side and PA on the other side. Previous studies indicated that the PA/PB1 interaction is crucial for IVs replication and that it is rather conserved between the various viral strains. Consequently, the inhibition of protein–protein interactions (PPIs) represents an excellent approach for the development of new antivirals. This strategy is based on the dissociation of viral protein complexes using various types of inhibitors, including small molecules that are able to bind at the interface between the viral subunits PA and PB1. In order to identify novel PA/PB1 interaction inhibitors, Kessler and colleagues screened a large library of compounds through an ELISA-based assay. Then, a plaque reduction assay on MDCK cells infected with IV A/WSN/33 (H1N1) was used to determinate the IC_50_ of the best performing compounds^24^. This strategy led to the identification of some benzoxadiazole derivatives (A–D) endowed with anti-influenza A virus activity in the micromolar concentration range (Figure 2). Nonetheless, these molecules displayed cytotoxic effects at relatively low concentrations^24^^,^^25^. In addition, the mode of action of the compounds was not completely validated, thus raising doubts as to whether the observed effects are a direct consequence of PA/PB1 interaction disruption.

Previously, our group has developed several 7-nitro-2,1,3-benzoxadiazole (NBD) derivatives as glutathione-*S*-transferase P1-1 (GSTP1-1) inhibitors^26–31^. The GSTP1-1 is an isoenzyme of the GST superfamily, which is selectively overexpressed in tumour cells and associated with the inhibition of apoptosis through its direct interaction with the mitogen-activated protein kinase (MAPK) named c-Jun-*N*-terminal Kinase (JNK1), and the scaffolding protein TNFα-receptor-associated-factor 2 (TRAF2)^32–34^. The prototype of our NBDs as GST inhibitors was the derivative 6-((7-nitrobenzo[*c*][1,2,5]oxadiazol-4-yl)thio)hexan-1-ol (NBDHEX) **1**, initially identified as a potent GSTP1-1 inhibitor^26^ although suffering from poor water solubility and low target selectivity over the isoform GSTM2-2, which is widely expressed in many non-cancerous tissues.

In an effort to obtain **1** derivatives with an improved pharmacological profile, we developed a library of more than 40 analogues of **1**. These include alkyl or alkoxyalkyl derivatives (**2**–**6**) possessing a free primary or secondary hydroxyl function, which is also esterified in compounds **7**–**11**. Compounds **12**, **14**, and **16**–**19** are propionic acid derivatives, while **13** and **15** possess an ethylamine side chain in which the nitrogen is either acetylated (**13**) or included in a piperidin-4-one ring (**15**). The library also includes compounds possessing small alkyl side chains (**20**, **21**), heteroaromatic groups (**22**, **23**), as well as aryl moieties variously functionalised at *ortho*, *meta*, and mainly *para* position (**24**–**42**). Finally, compounds **43**–**45** are analogues of **1** in which the sulphur in position 4 is oxidised to sulphinyl (**43**), sulphonyl (**44**) or replaced by an amino group (**45**)^27^.

**Figure 3.**
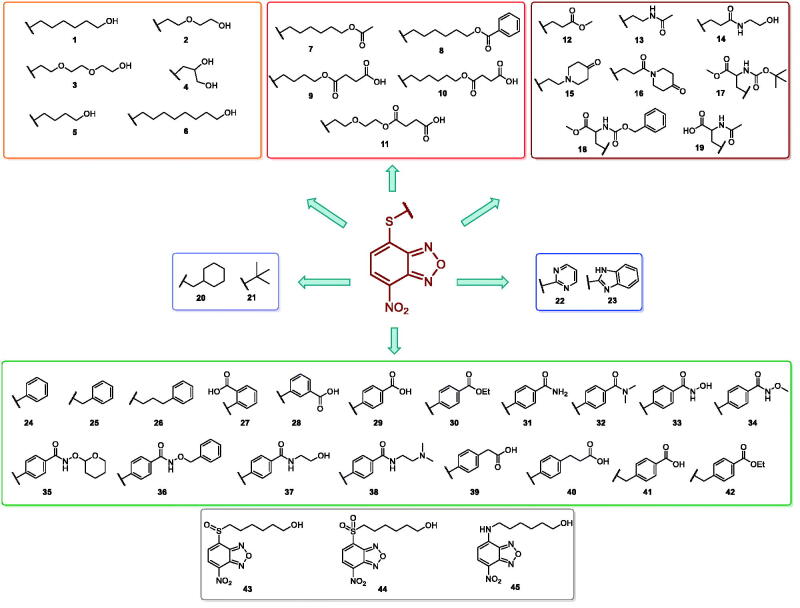
Structures of the compounds (**1**–**45**) evaluated in the study.

Since our GST inhibitors contain the same NBD central core of the compounds previously described as anti-IV molecules, we set out to evaluate derivatives **1**–**45** (Figure 3) as potential anti-IV agents to find derivatives possessing improved activity and/or selectivity and to draw preliminary structure–activity relationship (SAR) for this class of molecules.

## 2. Materials and methods

### 2.1. Chemistry

Unless otherwise specified, all chemicals used throughout this work were purchased from Sigma-Aldrich Srl (Milan, Italy). ^1^H-NMR spectra were recorded at 400 MHz on a Bruker AC 400 spectrometer (Bruker, Billerica, MA, USA); reporting chemical shifts in *δ* (ppm) units relative to the internal reference tetramethylsilane (Me_4_Si). All compounds were routinely checked by TLC and ^1^H-NMR. TLC was performed on aluminium-backed silica gel plates (Merck DC, Alufolien Kieselgel 60 F254, Kenilworth, NJ, USA) with spots visualised by UV light. Yields of all reactions refer to the purified products. All chemicals were of the highest purity. Mass spectra were recorded on an API-TOF Mariner by Perspective Biosystem; samples were injected by a Harvard pump using a flow rate of 5–10 µL/min, infused in the Electrospray system. Elemental analyses were performed by a PE 2400 (Perkin-Elmer, Waltham, MA) analyser and have been used to determine purity of the described compounds, which is >95%. Analytical results are within ±0.40% of the theoretical values.

Melting points were determined on a Buchi 530 melting point apparatus (Flawil, Switzerland) and are uncorrected.

#### 2.1.1. Synthesis of 4–(4-((7-nitrobenzo[c][1,2,5]oxadiazol-4-yl)thio)butoxy)-4-oxobutanoic acid (9)

Succinic anhydride (196 mg, 1.96 mmol, 2.0 eq.) was added to a solution of 6-((7-nitrobenzo[*c*][1,2,5] oxadiazol-4-yl)thio)hexan-1-ol (**5**) (260 mg, 0.98 mmol, 1 eq.) and 4-(dimethylamino)pyridine (DMAP) (120 mg, 0.98 mmol, 1.0 eq.) in dry dichloromethane (DCM) (9 ml) and the resulting mixture was stirred at reflux for 4 h. Upon completion, the reaction mixture was poured into a solution of 2 N HCl (22 ml) and extracted with ethyl acetate (4 × 25 ml). The organic phase was then washed with saturated sodium chloride solution (25 ml), dried over anhydrous sodium sulphate, filtered, and concentrated under vacuum. Finally, the crude residue was purified by silica gel column chromatography eluting with the appropriate mixture CHCl_3_/MeOH 25:1 to provide the final compound as a yellow powder. Yield: 70%. M.p.: 90–93 °C. ^1^H-NMR (DMSO) *δ* 1.809–1.838 (m, 4H, –SCH_2_*CH_2_CH_2_*CH_2_O–), 2.478–2.509 (*m*, 4H, –OCO*CH_2_CH_2_*COOH), 3.408 (*t*, 2H, –S*CH_2_*CH_2_CH_2_CH_2_O–), 4.100 (*t*, 2H, –SCH_2_CH_2_CH_2_*CH_2_*O–), 7.534 (d, 1H, C*H* benzoxadiazole ring), 8.574 (d, 1H, C*H* benzoxadiazole ring), 12.126–12.266 (br s, 1H, COO*H*). MS (ESI), *m/z*: 368.1 [M – H]^-^. Anal. (C_14_H_15_N_3_O_7_S) Calcd. (%): C, 45.53; H, 4.09; -N, 11.38; S, 8.68. Found (%) C, 45.68; H, 4.11; N, 11.30; S, 8.64.

#### 2.1.2. Synthesis of 4–(2–(2-((7-nitrobenzo[c][1,2,5]oxadiazol-4-yl)thio)ethoxy)ethoxy)-4-oxobutanoic acid (11)

Succinic anhydride (277.8 mg, 2.77 mmol, 3.0 eq.) was added to a solution of 2-(2-((7-nitrobenzo[*c*][1,2,5]oxadiazol-4-yl)thio)ethoxy)ethan-1-ol (264 mg, 0.93 mmol, 1 eq.) (**2**) and 4-(dimethylamino)pyridine (DMAP) (169.5 mg, 1.39 mmol, 1.5 eq.) in dry dichloromethane (DCM) (9 ml) and the resulting mixture was stirred at reflux for 4 h. Upon completion, the reaction mixture was poured into a solution of 2 N HCl (22 ml) and extracted with ethyl acetate (4 × 30 ml). The organic phase was then washed with saturated sodium chloride solution (30 ml), dried over anhydrous sodium sulphate, filtered, and concentrated under vacuum. Finally, the crude residue was purified by silica gel column chromatography eluting with the mixture CHCl_3_/MeOH 20:1 to provide the final compound. Yield: 51%. M.p.: 74–76 °C. ^1^H-NMR (DMSO) *δ* 2.461–2.509 (*m*, 4H, CO*CH_2_*C*H_2_*COOH), 3.598 (*t*, 2H, –S*CH_2_*CH_2_OCH_2_CH_2_O–), 3.673 (*t*, 2H, –SCH_2_CH_2_O*CH_2_*CH_2_O–), 3.835 (*t*, 2H, –SCH_2_*CH_2_*OCH_2_CH_2_O–), 4.148 (*t*, 2H, –OCH_2_*CH_2_*O–), 7.583 (d, 1H, C*H* benzoxadiazole ring), 8.579 (d, 1H, C*H* benzoxadiazole ring), 12.170–12.2174 (br s, 1H, COO*H*). MS (ESI), *m/z*: 384.1 [M - H]^-^. Anal. (C_14_H_15_N_3_O_8_S) Calcd. (%): C, 43.64; H, 3.92; N, 10.90; S, 8.32. Found (%) C, 43.80; H, 3.94; N, 10.82; S, 8.27.

#### 2.1.3. * General procedure for the synthesis of compounds 19, 22, 23* example: preparation of 4-nitro-7-(pyrimidin-2-ylthio)benzo[*c*][1,2,5]oxadiazole (22)

Pyridine (0.69 ml, 8.62 mmol, 3.5 eq.) and 2-mercaptopyrimidine (275.9 mg, 2.46 mmol, 1.0 eq.) were added to a solution of 4-chloro-7-nitrobenzo[*c*][1,2,5]oxadiazole (491.8 mg, 2.46 mmol, 1.0 eq.) in a mixture (18 ml) EtOH:H_2_O (0.3:1 *v/v*). The mixture was stirred at room temperature for 16 h. Subsequently, the suspension was filtered, and the crude solid was washed on the filter with H_2_O. The compound obtained was first purified by trituration with CHCl_3_ and then by column chromatography on SiO_2_ gel eluting with a mixture CHCl_3_/MeOH (90:1). Yield: 76%. M.p.: 144–147 °C. ^1^H-NMR (DMSO): *δ* 7.416 (*t*, 1H, *CH* pyrimidine ring), 8.288 (d, 1H, C*H* benzoxadiazole ring), 8.683–8.696 (*m*, 2H, C*H* pyrimidine ring), 8.719 (d, 1H, C*H* benzoxadiazole ring). MS (ESI), *m/z*: 276.0 [M + H]^+^. Anal. (C_10_H_5_N_5_O_3_S) Calcd. (%): C, 43.64; H, 1.83; N, 25.44; S, 11.65. Found (%) C, 43.76; H, 1.85; N, 25.37; S, 11.59.

##### 2.1.3.1. *N*-acetyl-*S*-(7-nitrobenzo[*c*][1,2,5]oxadiazol-4-yl)cysteine (19)

Yield: 78%. M.p.: 166–168 °C. ^1^H-NMR (DMSO): *δ* 1.857 (*s*, 3H, –NHCO*CH_3_*), 3.575–3.630 (dd, 1H, –S*CH*HCHNH–), 3.792–3.837 (dd, 1H, –S*C*H*H*CHNH–), 4.631–4.685 (*m*, 1H, CH_2_*CH*NH), 7.597 (d, 1H, C*H* benzoxadiazole ring), 8.540 (d, 1H, CH*NH*COCH_3_), 8.601 (d, 1H, C*H* benzoxadiazole ring), 13.107–13.271 (br s, 1H, COO*H*). MS (ESI), *m/z*: 325.0 [M - H]^-^. Anal. (C_11_H_10_N_4_O_6_S) Calcd. (%): C, 40.49; H, 3.09; N, 17.17; S, 9.83. Found (%) C, 40.60; H, 3.11; N, 17.11; S, 9.76.

##### 2.1.3.2. 4-((1*H*-benzo[d]imidazol-2-yl)thio)-7-nitrobenzo[*c*][1,2,5]oxadiazole (23)

Yield: 81%. M.p.: 206–208 °C. ^1^H-NMR (DMSO): *δ* 7.136 − 7.417 (br m, 2H, C*H* benzimidazole ring), 7.430 − 7.716 (*m*, 3H, C*H* benzimidazole and benzoxadiazole rings), 8.582–8.633 (d, 1H, C*H* benzoxadiazole ring), 13.475 (br s, N*H*). MS (ESI), *m/z*: 314.0 [M + H]^+^. Anal. (C_13_H_7_N_5_O_3_S) Calcd. (%): C, 49.84; H, 2.25; N, 22.35; S, 10.23. Found (%) C, 49.90; H, 2.27; N, 22.28; S, 10.17.

### 2.2. Cell cultures

Madin-Darby canine kidney (MDCK) cells and human lung epithelial cells (A549) were grown in RPMI 1640 and DMEM medium, respectively, supplemented with 10% foetal bovine serum (FBS), 0.3 mg/mL glutamine, 100 U/mL penicillin and 100 µg/mL streptomycin.

### 2.3. Cytotoxicity assay

The cytotoxicity of compounds was estimated on MDCK cells by using MTT [3–(4,5-dimethylthiazol-2-yl)-2,5-diphenyl tetrazolium bromide] (Sigma-Aldrich, St. Louis, MO, USA) assay. Briefly, cells were plated in a 96-well plate at a concentration of 2 × 10^4^/well in RPMI-1640 without phenol red, supplemented with 10% FBS. After 24 h plating, the compounds dissolved in DMSO (concentrations range 1–30 µM) were added and cells incubated for the following 18 h. Then the medium was replaced with 50 μL of a 1 mg/ml solution of MTT and cells were incubated at 37 °C for 3 h. Following incubation, the remaining water insoluble formazan was solubilised in absolute isopropanol containing 0.1 N HCl. Absorbance of converted dye was measured in an ELISA plate reader at the wavelength of 570 nm. The cytotoxicity of the compounds was calculated as the percentage of reduction of the viable cells compared with the drug-free control culture^35^.

In parallel, cell viability was estimated by Trypan Blue (0.02%) exclusion in MDCK and A549 cells treated or not with the compounds (concentrations range 1–30 µM) and incubated at 37 °C for the following 24 h. Trypan Blue exclusion analysis was also used to calculate CC_50_ values for selected compounds. CC_50_ was defined as the drug concentration required for reducing the viability of A549 cells by 50%.

### 2.4. Virus production and infection

Influenza virus A/Puerto Rico/8/34 H1N1 (PR8 virus) was grown in the allantoic cavities of 10-day-old embryonated chicken eggs. After 48 h at 37 °C, the allantoic fluid was harvested and centrifuged at 5000 rpm for 30 min to remove cellular debris. Confluent monolayers of epithelial cells (A549 or MDCK) were challenged for 1 h at 37 °C with PR8 at a multiplicity of infection (m.o.i.) of 0.001 incubated for 1 h at 37 °C, washed with PBS, and then incubated with medium supplemented with 2% FCS. Mock infection was performed with the same dilution of allantoic fluid from uninfected eggs^36^.

### 2.5. Haemagglutination (HAU) assay

MDCK cells plated at concentration of 2 × 10^5^/mL were infected with PR8 (0.001 m.o.i.) and, after the adsorption period, washed with PBS and treated with different concentrations (0–30 μM) of each compound. After 24 h post infection (p.i.), viral production was quantified in the supernatants of infected cells by measuring the haemagglutinin units (HAU), using human type 0 Rh + erythrocytes. Compounds were dissolved in DMSO to prepare stock solutions and diluted in RPMI to final concentrations of 0–30 µM. Compounds were added after the adsorption period and maintained in the culture media until the end of the experiments. Control cells were treated with DMSO alone at the same concentration present in the test substance being evaluated. The highest DMSO concentration present in the culture medium was 0.2%. The concentration of compound required to inhibit viral replication of 50% (IC_50_) was determined by regression analysis using Microsoft Excel software, considering untreated infected cells as control (100%). Experiments were performed in triplicates and IC_50_ values reported as ± standard deviation (s. d.).

### 2.6. In-Cell Western (ICW) assay

The ICW assay was performed using the Odyssey Imaging System (LI-COR, Lincoln, NE)^37^. A549 cells grown in 96-well plates (2 × 10^4^ cells/well), either infected or mock-infected (Ctr) with PR8, were fixed with 4% formaldehyde, washed, permeabilised with 0.1% Triton X-100 and incubated with PBS containing Odyssey Blocking buffer (LI-COR Biosciences, Lincoln, NE). The cells were then stained at 4 °C overnight with mouse anti-NP (1:400; Santa Cruz Biotechnology) together with Cell Tag (1:2000; LI-COR Biosciences, Lincoln, NE) in PBS containing 5% Odyssey Blocking Buffer. Cells were then washed and stained with goat anti-mouse IRDyeTM 800 antibodies (1:3000; LI-COR Biosciences, Lincoln, NE). Protein expression was quantified using the Odyssey Imaging System. For statistical analysis, integrated intensities of fluorescence in wells were determined using software provided with the imager station (LI-COR). The relative amount of NP protein was obtained by normalising to the Cell Tag in all experiments. Data obtained from mock infected A549 cells treated with selected compounds at different concentrations and stained with Cell Tag were used to calculate the CC_50_ values.

## 3. Results

### 3.1. Chemistry

**Scheme 1.**
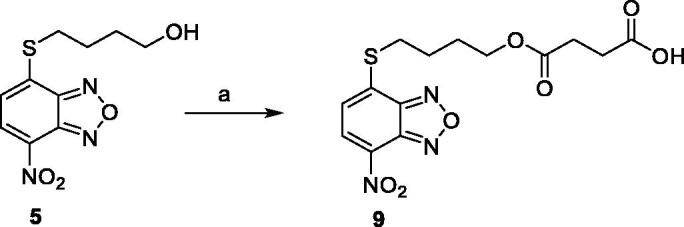
Compound **9** preparation. Reagents and conditions: (a) succinic anhydride, DMAP, dry DCM, reflux.

Compounds **1**^26^, **2–3**^38^, **4–6**^27^, **7–8**^39^, **10**^40^, **12**^27^,**13–18**^27^, **20–21**^27^, **24–43**^27^ have been prepared as described in previous reports. Compound **9** was prepared through a reaction between succinic anhydride and **5** in dry dichloromethane (DCM) in the presence of 4-(dimethylamino)pyridine (DMAP) under reflux conditions (Scheme 1).

**Scheme 2.**
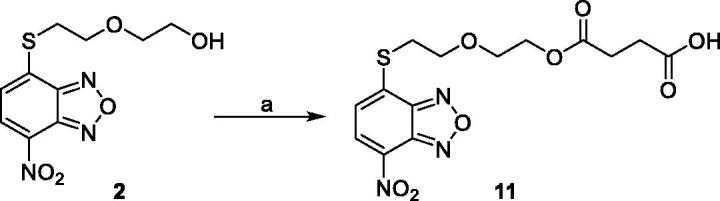
Reagents and conditions: (a) succinic anhydride, DMAP, dry DCM, reflux.

Compound **11** was prepared through a reaction between succinic anhydride and **2** in dry DCM in the presence of DMAP under reflux conditions (Scheme 2).

**Scheme 3.**
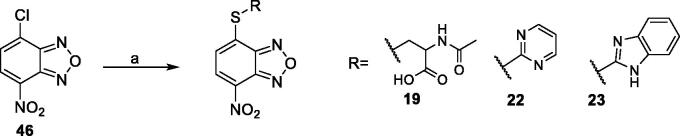
Preparation of compounds **19**, **22**, and **23**. Reagents and conditions: (a) appropriate thiol (R-SH), pyridine, EtOH:H_2_O (0.3:1 *v/v*), rt.

The derivatives **19, 22,** and **23** were prepared through a reaction between the commercially available 4-chloro-7-nitrobenzo[*c*][1,2,5]oxadiazole (**46**) and the proper commercial thiol in a mixture EtOH:H_2_O (0.3:1 *v/v*) in the presence of pyridine at room temperature (Scheme 3).

### 3.2. Cytotoxicity and antiviral activity of NBD derivatives in MDCK cells

In a first set of experiments, we assessed the potential cytotoxicity of the compounds. We plated MDCK at a concentration of 2 × 10^4^/well and, after 24 h, the cells were treated with various concentrations (range 1–30 µM) of each compound and incubated for the following 24 h. The cytotoxicity of each compound was assessed through a MTT assay, as described in the Materials and Methods section. In parallel, we estimated cell viability in the presence of each compound at the tested concentrations by Trypan Blue exclusion. Microscopic examination and Trypan Blue exclusion demonstrated that most compounds exerted a toxic effect at all tested concentrations and were therefore excluded for the antiviral activity screening (Table 1). Notably, compounds **8, 10, 11, 14, 19, 21, 23, 27, 28, 30, 31, 33, 37, 38, 39, 42, 43, 44,** and **45** were not toxic at any tested concentration, while compounds **9, 12, 17, 25, 40,** and **41** were toxic only at higher concentrations (starting from 20–30 μM).

**Table 1.** Antiviral activity and cytotoxicity of NBD derivatives **1–45**. 
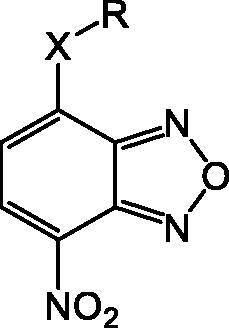

Compd	X	*R*	IC_50_ (µM)	Cytotoxicity
**1**	S	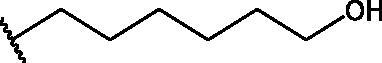	–	Toxic*^a^*
**2**	S	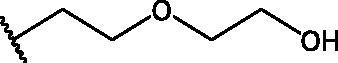	–	Toxic*^a^*
**3**	S	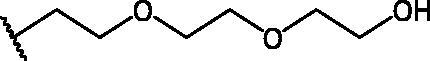	–	Toxic*^a^*
**4**	S	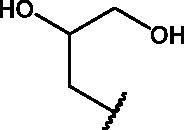	–	Toxic*^a^*
**5**	S	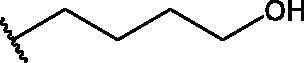	–	Toxic*^a^*
**6**	S	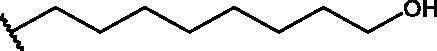	–	Toxic*^a^*
**7**	S	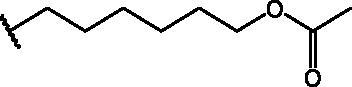	–	Toxic*^a^*
**8**	S	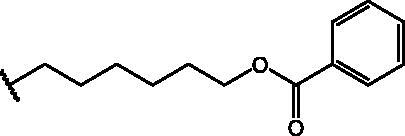	Inactive*^b^*	NOT toxic
**9**	S	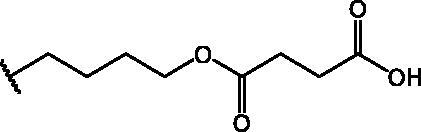	17.1 ± 0.9	Toxic from 30 µM
**10**	S	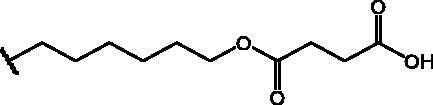	22.2 ± 1.4	NOT toxic
**11**	S	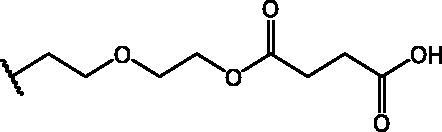	34.9 ± 3.6	NOT toxic
**12**	S	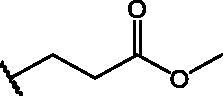	1.1 ± 0.1	Toxic from 20 µM
**13**	S	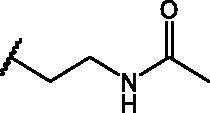	–	Toxic*^a^*
**14**	S	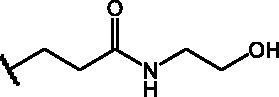	Inactive*^b^*	NOT toxic
**15**	S	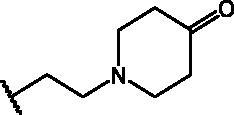	–	Toxic*^a^*
**16**	S	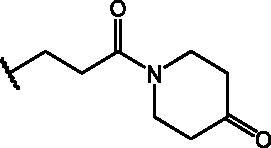	–	Toxic*^a^*
**17**	S	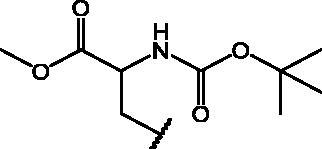	4.0 ± 0.4	Toxic from 20 µM
**18**	S	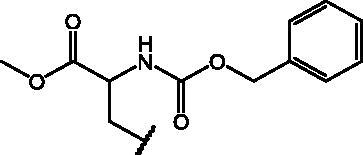	–	Toxic*^a^*
**19**	S	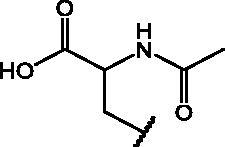	24.0 ± 2.3	NOT toxic
**20**	S	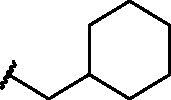	–	Toxic*^a^*
**21**	S	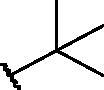	Inactive*^b^*	NOT toxic
**22**	S	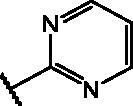	–	Toxic*^a^*
**23**	S	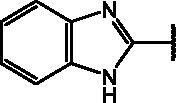	Inactive*^b^*	NOT toxic
**24**	S	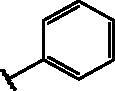	–	Toxic*^a^*
**25**	S	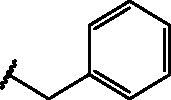	6.3 ± 0.5	Toxic from 20 µM
**26**	S	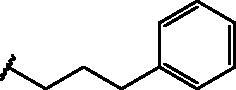	–	Toxic*^a^*
**27**	S	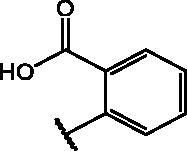	Inactive*^b^*	NOT toxic
**28**	S	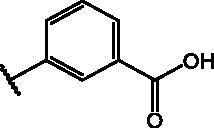	Inactive*^b^*	NOT toxic
**29**	S	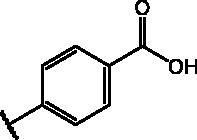	–	Toxic*^a^*
**30**	S	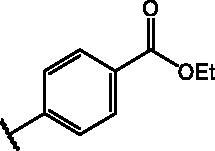	Inactive*^b^*	NOT toxic
**31**	S	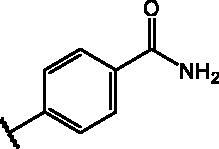	Inactive*^b^*	NOT toxic
**32**	S	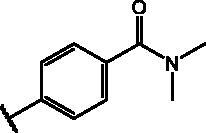	–	Toxic*^a^*
**33**	S	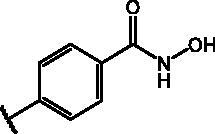	Inactive*^b^*	NOT toxic
**34**	S	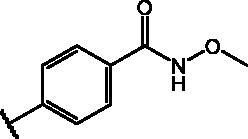	–	Toxic*^a^*
**35**	S	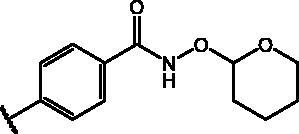	–	Toxic*^a^*
**36**	S	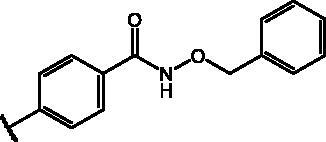	–	Toxic*^a^*
**37**	S	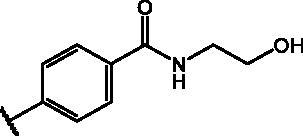	Inactive*^b^*	NOT toxic
**38**	S	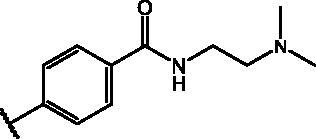	Inactive*^b^*	NOT toxic
**39**	S	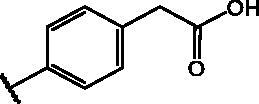	Inactive*^b^*	NOT toxic
**40**	S	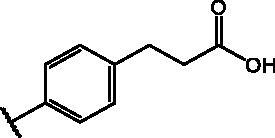	18.3 ± 1.6	Toxic from 25 µM
**41**	S	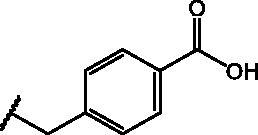	10.8 ± 0.6	Toxic from 20 µM
**42**	S	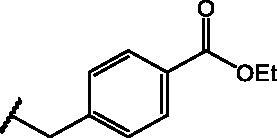	17.2 ± 1.6	NOT toxic
**43**	SO	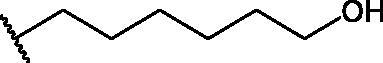	Inactive*^b^*	NOT toxic
**44**	SO_2_	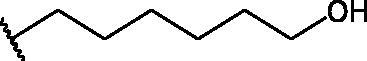	Inactive*^b^*	NOT toxic
**45**	NH	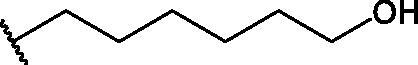	Inactive*^b^*	NOT toxic

*^a^*Toxic at all tested concentrations (1 − 30 µM) as evaluated by both MTT and Trypan Blue exclusion assays. *^b^*At maximum tested concentration (30 µM).

Based on cytotoxicity studies, MDCK cells were infected with influenza A/PR8 H1N1 virus strain and, after viral adsorption, cells were treated with different concentrations (ranging from 0.25 to 30 μM) of each compound. After 24 h post infection (p.i.), we quantified viral production through a haemagglutination assay (HAU) on the supernatants of infected cells. Compounds **9**, **11**, **12**, **17**, **19**, **25**, **40**, and **41** were effective against IV (Table 1 and Figure S1), as demonstrated by their IC_50_ (the concentration of compound required to inhibit viral replication of 50%). Compounds **6**, **8**, **14**, **21**, **23**, **27**, **28**, **30, 31**, **32**, **33**, **37**, **38**, **39**, **43**, **44**, and **45** did not inhibit viral replication up to the maximum tested dose (30 µM) and are indicated as inactive in Table 1.

### 3.3. Antiviral activity of compounds 12, 17 and 25 in A549 cells

Among the active molecules, **12, 17** and **25** were the only ones to display IC_50_ values below 10 µM, thus being the most effective compounds against IV. Therefore, we decided to evaluate their antiviral activity on a different cell line highly permissive to IVs, the human lung epithelial A549 cells. Viral replication measured by HAU assay demonstrated that all three compounds were effective against IV. In particular, the IC_50_ for **12** was 5.1 μM, while the CC_50_ calculated by counting the number of dead cells stained with Trypan Blue compared to the live cells was 54.5 μM. Hence, the Selectivity Index (SI = CC_50_/IC_50_) for compound **12** is equal to 11, suggesting that the molecule is active at concentrations that are far below its cytotoxicity threshold. The IC_50_ values for **17** and **25** were 11.7 and 1.9 μM, respectively, with the SIs equal to 2 and 6, respectively.

**Figure 4.**
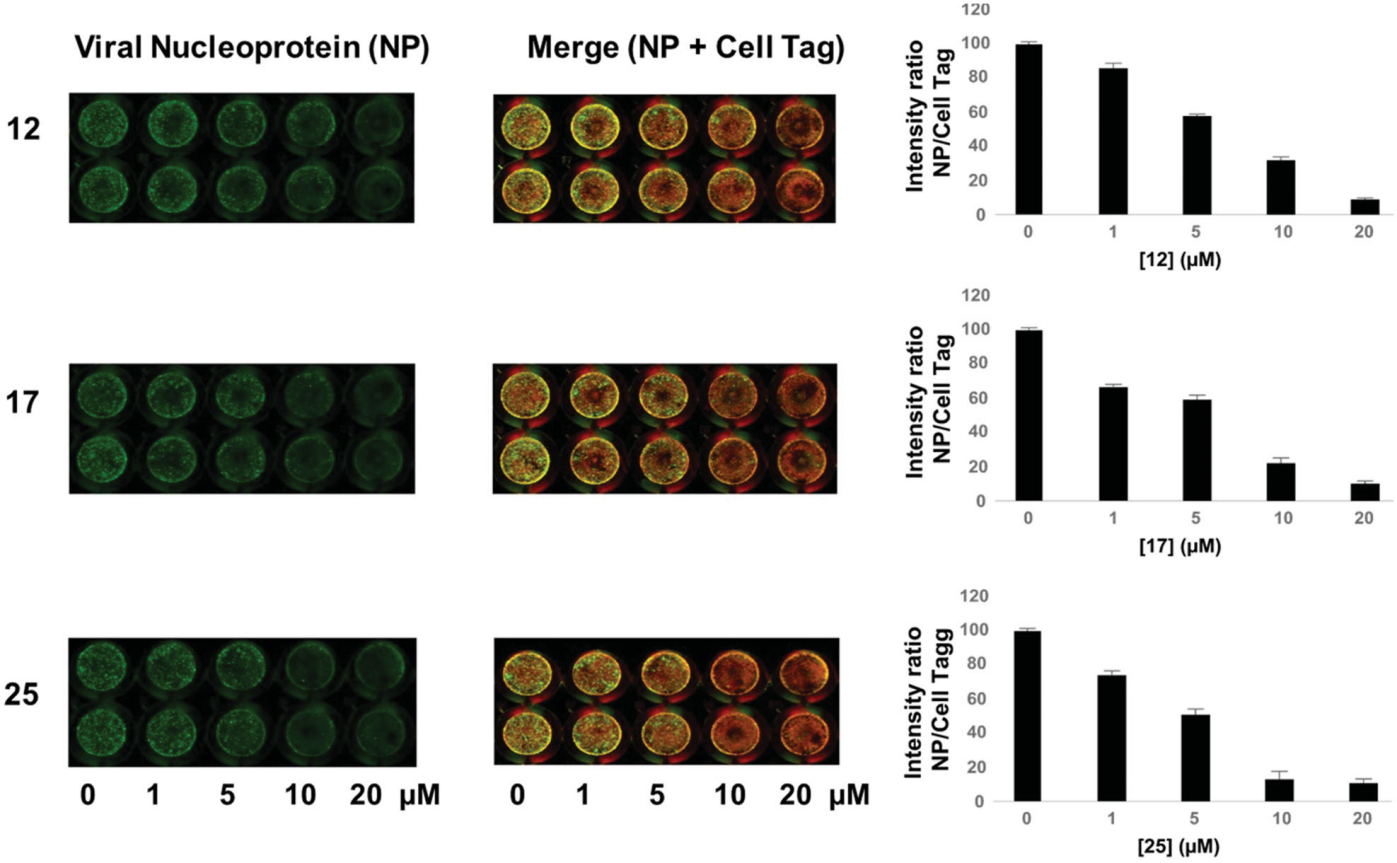
ICW assay on A549 cells infected with PR8 and treated with NBD derivatives **12**, **17** and **25** (concentration range 1–20 μM). *Left panel*: The integrity of cell monolayer was revealed by Cell Tag on the 680 nm channel (red); viral NP expression on the 800 nm channel (green); merged images show the overlapping between viral protein and infected cells (yellow). *Right panel*. Fluorescence intensities determined by the Odyssey software and the ratios betwen NP and Cell Tag signal were calculated and averaged for duplicate wells. The values are shown as a function of compounds concentration. Error bars indicate s. d. The percentage (%) of fluorescence intensity was calculated respect to untreated infected cells (considered as 100%).

Finally, we tested these compounds following a recently described method for viral titration^37^^,^^41^, the In Cell Western (ICW) assay. This approach is based on the evaluation of the viral nucleoprotein (NP) expression revealed by labelling cells with anti-NP antibodies. Briefly, infected confluent monolayers of A549, treated with increasing concentrations (1–20 μM) of the three compounds, were fixed and permeabilised (see Materials and Methods section), then incubated with anti-NP antibodies and with Cell Tag (a non-specific cell stain used to evaluate the integrity of cell monolayer). The fluorescence intensity of NP (green) and Cell Tag (red) was measured by Odyssey Imaging System. In parallel, mock infected A549 cells were treated with compounds **12**, **17,** and **25** and stained with Cell Tag to evaluate their toxicity and to calculate the CC_50_ using the ICW assay. As reported in Figure 4 (merge panel), the green fluorescence intensity released by viral NP labelled with specific antibodies indicated that NP expression was dose-dependently decreased in cells treated with the compounds compared to untreated ones. This is confirmed by the plots on the right panel of Figure 4, showing that the intesity ration between NP and Cell Tag decreases as compounds concentrations increase. The IC_50_ values obtained by ICW were in line with what already observed with the previous approach (IC_50_ for **12 **=** **10 μM, IC_50_ for **17 **=** **9.6 μM, and IC_50_ for **25 **=** **8.9 μM). Moreover, SI values calculated using the ICW IC_50_s and CC_50_s (Table 2) confirmed the selectivity of the three molecules.

**Table 2.** Antiviral activity and selectivity of compounds **12**, **17**, and **25** in A549 cells as determined by both HAU and ICW assays.

Compd	Structure	HAU – A549		ICW – A549	
		IC_50_ (µM)	SI^*a*^	IC_50_ (µM)	SI*^b^*
**12**	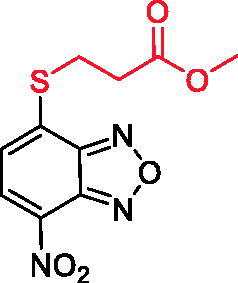	5.1 ± 0.2	11	10.0 ± 0.5	5
**17**	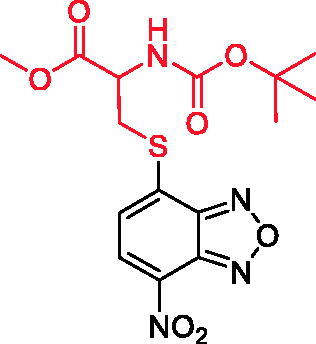	11.7 ± 1.0	2	9.6 ± 0.8	3
**25**	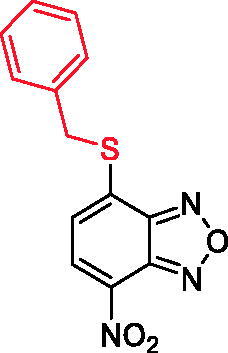	1.9 ± 0.1	6	8.9 ± 0.6	4

*^a^*SI: CC_50_/IC_50_ HAU – A549. *^b^*SI: CC_50_/IC_50_ ICW – A549.

## 4. Discussion

Over the past few years, many efforts have been made to obtain novel drugs for the treatment of infections caused by IVs. These endeavours resulted in the recent antivirals baloxavir marboxil and favipiravir both targeting IV RdRp with different modes of action. In an effort to find new anti-IV agents, we assessed our in-house library of NBD derivatives since they possess the same core of previously reported molecules endowed with anti-IV activity, although those compounds were cytotoxic at relative low concentrations and still necessitate validation of their mode of action^24^^,^^25^. We evaluated our compound library in two different cell lines infected by PR8, a strain of H1N1 influenza A virus.

The initial assay devoted to preliminary assessing the cytotoxicity of the compounds revealed that 25 molecules were not toxic in the concentration range we tested (1–30 µM) or toxic only at higher concentrations (≥20 µM) (Table 1). All 4-thioether-NBD derivatives possessing an alkyl/alkoxy alcohol (**1 − 6**) side chain were cytotoxic at all tested concentrations. Esterification of **1** with an acetyl group (**7**) did not change the toxicity profile; similarly, acylated or alkylated ethylamine derivatives **13** and **15** were cytotoxic.

Conversely, compound **8**, the benzoic ester of **1**, was not toxic at any tested concentration, but did not display any antiviral activity. Notably, esterification of **1**, **2**, and **5** with succinic acid yielding compounds **10**, **9**, and **11**, respectively, abolished the cytotoxicity and resulted in mild anti-IV activity. Among the propionic acid derivatives, only two compounds were cytotoxic, namely the piperidin-4-one amide derivative **16** and compound **18**, bearing a Cbz-protected amino group in C2. Among the non-toxic molecules, the methyl propionate derivatives **12** and **17** displayed the best antiviral activity, while **19**, bearing a free carboxylic acid, showed mild antiviral properties and the hydroxyethyl amide derivative **14** was inactive. Compounds **20** and **21** were toxic and inactive, respectively, suggesting that purely alkyl side chains may be detrimental for antiviral activity. Similarly, compounds bearing an aromatic moiety directly linked to the sulphur were either toxic or substantially inactive. Moreover, compounds **29** and **39**, bearing a benzoic or phenylacetic moiety were toxic and inactive, respectively. Remarkably, benzyl derivatives **25**, **41** and **42** were not cytotoxic and possessed good anti-IV activity, suggesting that the presence of a methylene spacer between the sulphur and the side-chain aromatic group is essential for activity and selectivity. Finally, **1** analogue in which the sulphur at position 4 was replaced by a 4-sulphinyl (**43**), 4-sulphonyl (**44**) or 4-amino (**45**) were not toxic, but did not possess any antiviral activity at the tested concentrations. These data suggest that the presence of a reduced sulphur is pivotal for antiviral activity.

Overall, 10 compounds of the library possessed antiviral activity, all of them being 4-thioether derivatives of NBD. The active molecules can be divided into three subgroups: (i) the emisuccinic esters **9**, **10**, **11**; (ii) the propionic acid derivatives **12**, **17,** and **19**; (iii) the 4-benzylthio derivatives **25**, **41, 42**. In group (i), it is apparent that increasing the length of the alkyl chain reduces the antiviral activity as demonstrated by the increased IC_50_ value of **10** compared to **9**. In addition, the introduction of polar atoms, such as oxygen, is detrimental for compound activity as indicated by the higher IC_50_ value of **11** compared to **9**. In group (ii), methylation of the carboxylic acid function of *N*-α protected cysteine residues decreases the IC_50_ value at least six-fold (compare **17** with **19**), and the subsequent removal of the protected amino group at α position leads to a further increase of antiviral potency (compare **12** with **17**) suggesting that a small methyl propionate side chain is important for compound activity. In group (iii), the introduction of both carboxy (**41**) and carbethoxy (**42**) groups determine a decrease in antiviral activity compared to **25**, indicating that substitutions with polar moieties at *para* position are unfavourable for the 4-benzylthio-NBD derivatives series. Overall, the present data suggest that compact, hydrophobic substitutions are favourable for antiviral activity, alternatively benzyl derivatives are promising anti-IV compounds, although different substitutions in the benzene ring need to be explored.

Given their promising activity in MCDK cells, we assessed compounds **12**, **17**, and **25** also in the A549 cell line. The HAU and ICW assays performed on these cells infected with PR8 confirmed the antiviral activity and selectivity of the three molecules. Overall, compound **25** resulted as the most active in A549 cells, while **12** was the best one in MCDK cells and displayed the best selectivity profile in both cell lines (Table 2).

Despite their specific mode of action has not been clarified yet, the results of this study demonstrate that some 4-thio-NBD derivatives are effective against IV and shed light on structure–activity relationship that could be exploited for the design of new molecules. Indeed, compounds **12** and **25** with their simple structures amenable for modifications represent promising hit compounds for future medicinal chemistry optimisation studies.

In conclusion, we hope that the present study might drive the search to the identification and characterisation of new potential compounds for fighting IV.
